# Volume–outcome relationships in open and endovascular repair of abdominal aortic aneurysm: administrative data 2006–2018

**DOI:** 10.1002/bjs.11919

**Published:** 2021-05-26

**Authors:** T Tong, A Aber, J Chilcott, P Thokala, S J Walters, R Maheswaran, S Nawaz, S Thomas, J Michaels

**Affiliations:** 1 School of Health and Related Research, University of Sheffield, Sheffield, UK; 2 Sheffield Vascular Institute, Sheffield Teaching Hospitals, Sheffield, UK

## Abstract

**Background:**

The aim of this study was to use recent evidence to investigate and update volume–outcome relationships after open surgical repair (OSR) and endovascular repair (EVAR) of abdominal aortic aneurysm in England.

**Methods:**

Hospital Episode Statistics (HES) data from April 2006 to March 2018 were obtained. The primary outcome was in‐hospital death. Other outcomes included duration of hospital stay, readmissions within 30 days, and critical care requirements. Case‐mix adjustment included age, sex, HES year, deprivation index, weekend admission, mode of admission, type of procedure and co‐morbidities.

**Results:**

Annual volume of all repairs combined appeared to be an appropriate measure of volume. After case‐mix adjustment, a significant relationship between volume and in‐hospital mortality was seen for OSR (*P* < 0·001) but not for EVAR (*P* = 0·169 for emergency and *P* = 0·363 for elective). The effect appeared to extend beyond 60 repairs per year to volumes above 100 repairs per year. There was no significant relationship between volume and duration of hospital stay or 30‐day readmissions. In patients receiving emergency OSR, higher volume was associated with longer stay in critical care.

**Conclusion:**

Higher annual all‐procedure volumes were associated with significantly lower in‐hospital mortality for OSR, but such a relationship was not significant for EVAR. There was not enough evidence for a volume effect on other outcomes.

## Abstract (es)

**Antecedentes:**

El objetivo de este estudio fue, utilizando los datos más recientes, analizar y actualizar la relación entre el volumen quirúrgico y los resultados en la reparación abierta (*open surgical repair*, OSR) o endovascular (*endovascular repair*, EVAR) del aneurisma aórtico abdominal en Inglaterra.

**Métodos:**

Los datos se obtuvieron del *Hospital Episode Statistics* (HES) entre abril del 2006 y marzo del 2018. La variable principal fue la mortalidad hospitalaria. Se analizaron otras variables como la duración de la estancia hospitalaria (*length of stay*, LOS), la tasa de reingresos en los primeros 30 días y la necesidad de cuidados intensivos. Se realizó un ajuste de la casuística mediante edad, sexo, año, índice de deprivación, ingreso en fin de semana, forma de ingreso, tipo de procedimiento y comorbilidades.

**Resultados:**

El volumen anual de todas las reparaciones, independientemente de la técnica utilizada, puede ser considerada una medida de volumen adecuada. Tras el ajuste de casos, se observó una relación significativa entre volumen y mortalidad intrahospitalaria en la OSR (*P* < 0,01) pero no para la EVAR (*P* = 0,169 para cirugía de urgencia y 0,363 para cirugía electiva). Este efecto pareció hacerse extensivo pasadas las 60 reparaciones por año a volúmenes de más de 100 reparaciones anuales. No hubo una relación significativa entre el volumen y estancia hospitalaria o reingresos a los 30 días. Se halló una asociación entre mayor volumen y mayor duración de la estancia en cuidados intensivos tras la OSR de urgencia.

**Conclusión:**

Hubo una asociación significativa entre el mayor volumen de casos y menor mortalidad hospitalaria para la OSR, pero esta relación no fue significativa para la EVAR. No hubo pruebas suficientes del efecto de volumen en otros resultados estudiados.

This study used recent evidence to investigate and update volume–outcome relationships following open surgical repair (OSR) and endovascular repair (EVAR) of abdominal aortic aneurysm in England. Higher annual all‐procedure volumes were associated with significantly lower in‐hospital mortality for OSR, but such a relationship was not significant for EVAR. There was not enough evidence for a volume effect on other outcomes.

Higher volumes associated with lower mortality for open repair but not EVAR

## Introduction

There is variation in abdominal aortic aneurysm (AAA) care internationally, especially regarding AAA size thresholds for offering repair, and availability and use of endovascular options to treat AAA. A recent review^1^ of European studies that examined the AAA volume–outcome relationship found that most studies demonstrated improved outcomes with larger volumes. However, the definition of volume differed between studies^2–19^. Evidence from such studies has informed clinical guidelines that advocate minimum AAA volume thresholds^20–22^.

There are several concerns, however, with the evidence from previous studies and the implications for minimum volume. The data used in most of these studies were from the first decade of the current millennium when most elective AAA repairs were undertaken using open surgical repair (OSR). Some studies using recent data have not investigated the type of volume (such as endovascular aneurysm repair (EVAR) *versus* OSR, elective *versus* all repairs) in more detail to identify the most relevant measure of volume and its impact on outcomes^16^. Current data show that there has been a major shift, and that most elective AAA repair is performed using EVAR, whereas OSR remains the main method of repair for ruptured AAA^16^^,^^23^. This on its own warrants further investigation into the volume–outcome relationship to identify the impact of different definitions and thresholds for volume on the outcomes of elective and emergency AAA repair. A further concern is that previous studies investigating the volume–outcome relationship for EVAR from Hospital Episode Statistics (HES) data in the UK used approximate methods to identify and distinguish EVAR from OSR. This was because no EVAR‐specific codes were available before late 2005, which constitutes most of the time intervals covered in previous studies^4^^,^^5^^,^^17^.

The purpose of this study was to evaluate the impact of volume on outcomes in AAA surgery using more recent evidence from HES and improved methods. The impact of different definitions of volume on the results was examined. The volumes used to examine outcomes in this study included all repairs, elective repairs, emergency repairs, all EVAR and all OSR. The outcomes measured were in‐hospital mortality, duration of hospital stay, 30‐day readmissions and use of critical care in the index admission. The study reports on all repairs of infrarenal AAA from April 2006 to March 2018.

## Methods

HES patient care data with linkages to national mortality data from 1 April 2006 to 31 March 2018 were acquired. Inpatient episodes for patients with AAA were extracted. The episodes were then sorted chronologically and grouped into continuous inpatient stays (CIPS). The CIPS was used to define a hospital admission for volume–outcome analyses. The index admission was defined as the admission where patients received their first AAA repair. The methods used to identify the different case‐mix groups, including elective, emergency intact and ruptured AAA, have been reported in previous studies^24^^,^^25^. The primary outcome was in‐hospital death defined by whether the patient was discharged alive or dead at the end of the index admission. Other outcomes included duration of hospital stay and use of critical care in the index admission, and readmissions within 30 days of discharge. The critical care data were available only from 1 April 2008, so analysis of critical care use was restricted to a subset of the total cohort from 1 April 2008 to 31 March 2018. A validation study was conducted to compare the estimates from HES data with those from the National Vascular Registry^23^ (*Appendix**S1*, supporting information).

### Identification of abdominal aortic aneurysm repair sites

For each HES year in the data set, the volume per hospital for the different types of AAA repair was calculated. The hospitals performing AAA repair were identified using provider and treatment site codes as well as the postcode of the treatment site. These codes were used to account for any variation in the data because of movement of sites or change in provider name. The problem of using the provider code only was that one provider could have multiple sites that provided vascular services over the years. A unique classification system was developed to accurately identify vascular sites at which AAA repairs were performed over the study period.

### Definition of volume

It was necessary to account for the observed time trend in data on in‐hospital mortality (towards lower mortality in more recent years) in the volume–outcome analysis. In addition, because of the substantial service reconfigurations that took place during the study interval, annual volume was used for each specific year rather than average annual volume across the years. In this way, the same vascular unit providing AAA repair could be observed to have different volumes across the years. The main volume measure comprised all repairs (including both complex and infrarenal repairs). Several alternative definitions of volume were tested, based on counting elective repairs only, emergency repairs only, OSR only and EVAR only.

### Statistical analysis

The patient cohort was divided into four groups: emergency EVAR, emergency OSR, elective EVAR and elective OSR. The emergency groups included all patients with ruptured AAA and those admitted as emergency (determined from code for admission). Volume data are presented by quintiles. The data were divided into five equal portions so that each data group contained a similar number of observations. The short‐term outcomes were then summarized for each data quintile. Using the first quintile as the reference for comparison with the other quintiles, the χ^2^ test was used to investigate the impact of volume on binary outcomes (mortality and 30‐day readmission) and the Mann–Whitney–Wilcoxon test for continuous outcomes (duration of hospital stay and critical care stay). Baseline differences between data quintiles are presented in *Table S1* (supporting information).

The volume and in‐hospital mortality relationship was first investigated without adjusting for confounding factors. Then logistic regression models were used to adjust for age, sex, HES year of data, deprivation index, weekend admission, mode of admission, type of procedure and co‐morbidities. Patient co‐morbidities were identified using a modified version of the Charlson co‐morbidity categories^24^^,^^26^. In‐hospital mortality was modelled using fixed‐effect logistic regression analyses. A two‐stage process was employed to decide which co‐variables should be included in the final models. First, a comprehensive list of all possible variables was developed with input from vascular clinicians. Using this list, models were then fitted using a forward stepwise approach to understand the impact of each variable. The results were presented to a group of clinicians to discuss clinical validity. Afterwards, the co‐variables for the final models were decided (with group consensus). Hospital volume was included in the final model to determine the adjusted volume–outcome relationship. Models without the volume co‐variables were also used to calculate the standardized mortality ratio (SMR) and plot the adjusted volume–outcome relationship (indirect standardization). A multilevel modelling approach was also undertaken to confirm that it would not significantly change the results from the single‐level multivariable models (*Appendix**S2*, supporting information). R version 3.4.1 (R Foundation for Statistical Computing, Vienna, Austria) was used for statistical analysis.

## Results

Between April 2006 and March 2018, a total of 72 022 patients had AAA repairs. The largest group comprised 28 656 patients who underwent elective EVAR (39·8 per cent). Some 21 694 patients had elective OSR (30·1 per cent), 15 953 had emergency OSR (22·2 per cent), and the smallest group included 5719 patients who had emergency EVAR (7·9 per cent). *Table* *1* summarizes the characteristics of each clinical group.

**Table 1:** Patient summary

	Elective			Emergency		
	EVAR	OSR	All elective	EVAR	OSR	All emergency
**2006–2007 to 2017–2018**						
No. of procedures	28 656 (39·8)	21 694 (30·1)	50 350 (69·9)	5719 (7·9)	15 953 (22·2)	21 672 (30·1)
Age (years)*	75·5(7·2)	71·2(8·2)	73·7(7·9)	76·3(8·6)	73·5(8·9)	74·2(8·9)
Men (%)	88·9	85·9	87·6	84·0	82·3	82·7
In‐hospital death (%)	1·3	5·2	2·9	12·6	31·4	26·4
Duration of hospital stay (days)^†^	3 (2–5)	8 (6–12)	5 (2–8)	6 (3–13)	11 (6–22)	10 (4–19)
Readmission within 30 days (%)^‡^	15·2	11·6	13·7	23·4	15·9	18·3
**2008–2009 to 2017–2018** ^§^						
No. of procedures	26 548 (44·4)	15 693 (26·3)	42 241 (70·7)	5373 (9·0)	12 515 (20·3)	17 515 (29·3)
Duration of critical care stay (days)^†^	0 (0–1)	2 (1–4)	1 (0–2)	1 (0–2)	3 (0–7)	2 (0–5)
Duration of critical care stay (h)^†^	0 (0–23·0)	47·1 (19·4–94·2)	16·9 (0–47·1)	17·0 (0–51·7)	65·5 (0–162·4)	45·0 (0–127·2)

Values in parentheses are percentages unless indicated otherwise; values are

*mean(s.d.) and

^†^median (i.q.r.).

^‡^Percentages based on those who survived the index admission.

^§^Critical care data were available only from 2008–2009. EVAR, endovascular aneurysm repair; OSR, open surgical repair.

**Fig. 1:**
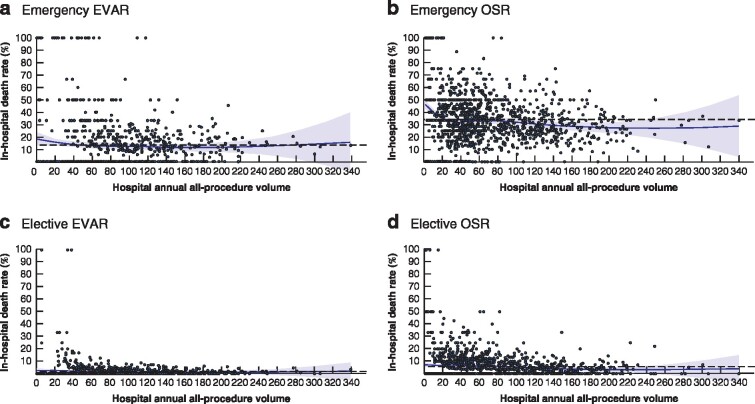
All‐procedure annual volume in relation to in‐hospital mortality **a** Emergency endovascular aneurysm repair (EVAR), **b** emergency open surgical repair (OSR), **c** elective EVAR and **d** elective OSR. The curve represents the approximate observed relationship between in‐hospital mortality rate and hospital annual abdominal aortic aneurysm repair volume. Each dot represents the mortality rate at a hospital in a specific year. The dashed horizontal line represents the mean for the whole cohort.

There was a total of 150 unique hospital sites (differentiated by postcodes) which performed at least five AAA repairs per year within the study interval. Through the time span, some sites were only active in a few years owing to service reconfiguration, and some new sites appeared. The number of active sites was 136 in 2006–2007 and 68 in 2017–2018. The total annual volume (including all complex and infrarenal repairs) of procedures at each site was counted for each HES year. *Table* *2* shows a summary of outcomes by data quintiles and clinical groups. *Fig*. *1* illustrates the relationship between annual volume and in‐hospital mortality at individual‐hospital level.

**Table 2:** Summary of volumes and outcomes by data quintiles, 2006–2007 to 2017–2018

Data quintile	Annual volume range (all procedures)	No. of sites^†^	No. of patients	Men (%)	Mean age (years)	Duration of hospital stay (days)*	*P* ^§^	In‐hospital death (%)	*P* ^¶^	Readmission (%)^‡^	*P* ^#^
**Emergency EVAR**											
1st	1–67	391	1170	84·3	76·3	10 (18·0)	–	13·3	–	24·4	–
2nd	68–103	175	1193	85·2	75·9	9 (17·1)	0·552	12·7	0·625	23·9	0·806
3rd	104–131	109	1113	86·3	76·8	8 (15·8)	< 0·001	12·9	0·731	23·6	0·695
4th	132–175	96	1100	82·2	76·6	9 (16·8)	0·024	10·6	0·041	20·8	0·060
5th	176–339	61	1143	82·0	75·8	9 (17·2)	0·124	13·7	0·777	24·3	0·992
**Emergency OSR**											
1st	1–40	588	3247	82·2	73·4	13 (21·2)	–	34·7	–	15·0	–
2nd	41–63	270	3171	82·4	73·9	12 (20·2)	0·234	33·3	0·245	15·0	0·997
3rd	64–97	203	3203	82·4	73·7	12 (20·5)	0·262	33·5	0·305	16·9	0·083
4th	98–138	160	3206	82·3	73·5	12 (21·5)	0·755	28·7	< 0·001	16·0	0·348
5th	139–339	138	3126	82·1	72·9	13 (23·3)	0·017	26·6	< 0·001	16·5	0·168
**Elective EVAR**											
1st	1–59	439	5795	89·0	75·4	4 (6·0)	–	1·5	–	14·1	–
2nd	60–92	209	5731	89·0	75·6	4 (5·8)	< 0·001	1·2	0·120	16·7	< 0·001
3rd	93–126	139	5825	89·2	75·6	3 (6·0)	< 0·001	1·3	0·326	15·9	0·010
4th	127–165	106	5584	89·0	75·6	4 (5·5)	< 0·001	1·2	0·098	14·4	0·669
5th	166–339	77	5721	88·3	75·4	3 (5·6)	< 0·001	1·2	0·103	14·8	0·303
**Elective OSR**											
1st	1–39	503	4344	86·0	71·5	9 (14·4)	–	6·3	–	11·0	–
2nd	40–62	282	4373	85·0	71·4	9 (12·7)	< 0·001	6·1	0·695	12·1	0·138
3rd	63–101	227	4349	85·8	71·4	8 (12·7)	< 0·001	5·9	0·521	12·1	0·138
4th	102–140	152	4358	86·1	71·2	8 (12·1)	< 0·001	4·0	< 0·001	10·8	0·716
5th	141–339	130	4270	86·5	70·4	8 (12·2)	< 0·001	3·7	< 0·001	11·9	0·221

*Values are median (mean).

^†^One site could be observed multiple times (different years).

^‡^Readmission within 30 days of discharge for those who survived the index admission. EVAR, endovascular aneurysm repair; OSR, open surgical repair.

^§^Comparison of hospital stay *versus* first quintile (Mann–Whitney–Wilcoxon test);

^¶^comparison of in‐hospital death *versus* first quintile (χ^2^ test);

^#^comparison of readmission *versus* first quintile (χ^2^ test).

Higher annual volume of AAA repair (all procedures) was significantly associated with lower in‐hospital mortality after OSR (both elective and emergency); there was a trend with increasing benefit beyond the current recommended threshold of 60 repairs per year, which appeared to extend beyond 100 repairs per year. There was no statistically significant relationship between volume and in‐hospital mortality for EVAR (both elective and emergency).

A consistent volume–outcome relationship was not observed for duration of hospital stay or 30‐day readmissions, although volume was associated with a slightly shorter hospital stay for elective operations above a volume of about 100 repairs per year. A statistically significant relationship between volume and duration of critical care stay in index admissions was observed for the emergency OSR group: higher volume was associated with higher critical care. Such a relationship was not observed in other case‐mix groups (*Table* *3*).

**Table 3:** Summary of volumes and critical care stay by data quintiles, 2008–2009 to 2017–2018

Data quintile	Annual volume range (all‐procedure volume)	No. of sites^†^	No. of patients	Men (%)	Mean age (years)	Duration of critical care (days)*	Duration of critical care (h)*	*P* ^‡^
**Emergency EVAR**								
1st	1–67	347	1084	84·1	76·5	0 (2·6)	6·1 (62·2)	–
2nd	68–103	151	1071	85·0	76·2	1 (2·7)	19·3 (64·2)	0·177
3rd	104–131	104	1100	86·1	76·8	0 (2·3)	11·8 (54·6)	0·856
4th	132–175	88	1056	82·3	76·7	0 (2·5)	11·1 (61·3)	0·771
5th	176–339	54	1062	81·6	75·9	1 (3·7)	22·5 (88·4)	< 0·001
**Emergency OSR**								
1st	1–44	452	2538	81·7	73·5	2 (5·8)	51·6 (140·7)	–
2nd	45–70	215	2361	83·0	74·0	2 (6·0)	58·0 (145·6)	0·068
3rd	71–106	156	2468	82·8	73·7	3 (5·9)	69·0 (143·4)	< 0·001
4th	107–141	121	2390	82·0	73·4	3 (6·4)	71·2 (154·5)	< 0·001
5th	142–339	113	2385	82·5	72·9	3 (6·7)	66·3 (161·4)	< 0·001
**Elective EVAR**								
1st	1–61	368	5451	89·2	75·5	0 (1·0)	0 (23·6)	–
2nd	62–94	171	5195	88·9	75·7	0 (0·8)	0 (19·1)	0·620
3rd	95–126	119	5304	89·4	75·7	0 (0·7)	0 (17·0)	0·056
4th	127–166	99	5371	88·8	75·6	0 (0·8)	0 (17·7)	0·301
5th	167–339	66	5227	88·4	75·5	0 (0·9)	0 (22·2)	< 0·001
**Elective OSR**								
1st	1–42	394	3152	85·7	71·1	2 (3·5)	45·8 (83·5)	–
2nd	43–71	239	3154	85·6	71·2	2 (3·2)	45·9 (75·6)	0·372
3rd	72–109	168	3252	87·1	71·0	2 (3·4)	47·8 (82·3)	< 0·001
4th	110–145	115	3021	87·7	70·5	2 (3·4)	47·5 (79·9)	< 0·001
5th	146–339	102	3114	87·5	70·1	2 (3·5)	45·3 (82·3)	0·910

*Values are mean (median).

^†^One site could be observed multiple times (different years). EVAR, endovascular aneurysm repair; OSR, open surgical repair.

^^‡^^Comparison of critical care stay *versus* first quintile (Mann–Whitney–Wilcoxon test).

### Case‐mix‐adjusted results

**Fig. 2:**
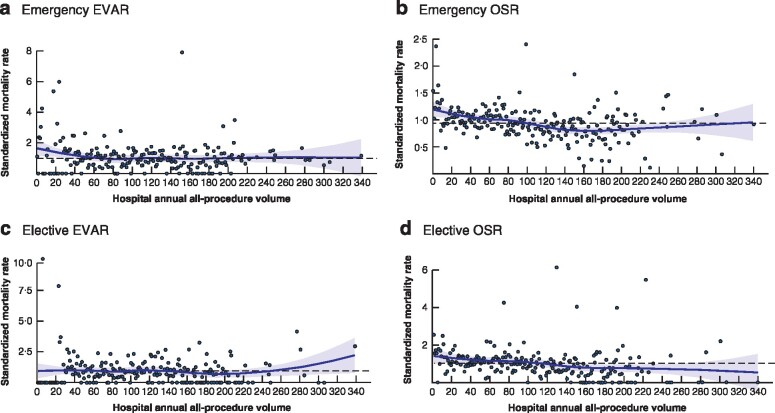
All‐procedure annual volume in relation to adjusted mortality **a** Emergency endovascular aneurysm repair (EVAR), **b** emergency open surgical repair (OSR), **c** elective EVAR and **d** elective OSR. The curve represents the approximate observed relationship between standardized mortality rate and hospital annual abdominal aortic aneurysm repair volume. Each dot represents the standardized mortality rate at a hospital in a specific year. The dashed horizontal line represents the mean for the whole cohort.

*Fig*. *2* shows the relationship between annual all‐procedure volume and adjusted mortality. Regression models were fitted for the four case‐mix groups to adjust for factors that may influence in‐hospital mortality. The adjusted relationship between volume and in‐hospital mortality is reflected by the coefficient of the volume co‐variable in each model (*Tables S2–S5*, supporting information). Statistically significant relationships between volume and in‐hospital mortality were seen for emergency OSR (odds ratio (OR) 0·997, 95 per cent c.i. 0·996 to 0·998; *P* < 0·001) and elective OSR (OR 0·996, 0·995 to 0·998; *P* < 0·001). However, there was no significant relationship for emergency EVAR (OR 0·999, 0·998 to 1·000; *P* = 0·169) or elective EVAR (OR 0·999, 0·997 to 1·001; *P* = 0·363). OR values of less than 1·00 indicate a negative association between volume and in‐hospital mortality. The negative relationship means that, as volume increases, the odds of in‐hospital death decrease by a factor equal to the OR. A more intuitive illustration of these results is provided in *Appendix**S3* (supporting information). The results from an alternative multilevel approach generally agreed with those from the single‐level approach reported above (*Appendix**S2*, supporting information).

### Impact of different volume definitions on outcome

The impact of different volume definitions on the relationship between volume and in‐hospital mortality was analysed by changing the definition of the volume co‐variable in each regression model from all repairs to elective‐only and emergency‐only repair, and open or endovascular procedures. Different definitions of volume affected the volume–outcome relationship (*Table* *4*). The relationship between volume and in‐hospital mortality after emergency OSR was statistically significant across all definitions of volume. The volume–outcome relationship following elective OSR was statistically significant across all definitions of volume except EVAR‐specific volume, although higher EVAR volume was still associated with lower OSR mortality but the *P* value was 0·060. The volume–outcome relationship following elective EVAR was not statistically significant across all definitions of volume. This suggests an absence of evidence for such a relationship. The volume–outcome relationship after emergency EVAR was not statistically significant across all definitions of volume, except for OSR‐specific volume; there was a significant association between higher OSR volume and lower EVAR mortality (*P* = 0·033).

**Table 4:** Changes in coefficient of volume co‐variable owing to different volume definitions

Volume measure	Case‐mix group group	Coefficient of volume co‐variable	Odds ratio	*P*
All procedures	Emergency EVAR	–0·00095	0·999 (0·998, 1·000)	0·169
	Emergency OSR	–0·00313	0·997 (0·996, 0·998)	< 0·001
	Elective EVAR	–0·00085	0·999 (0·997, 1·001)	0·363
	Elective OSR	–0·00362	0·996 (0·995, 0·998)	0·000
All elective procedures	Emergency EVAR	–0·00113	0·999 (0·997, 1·001)	0·237
	Emergency OSR	–0·00420	0·996 (0·995, 0·997)	< 0·001
	Elective EVAR	–0·00144	0·999 (0·996, 1·001)	0·268
	Elective OSR	–0·00503	0·995 (0·993, 0·997)	< 0·001
All emergency procedures	Emergency EVAR	–0·00349	0·997 (0·992, 1·001)	0·103
	Emergency OSR	–0·00918	0·991 (0·989, 0·993)	< 0·001
	Elective EVAR	–0·00094	0·999 (0·993, 1·005)	0·744
	Elective OSR	–0·00970	0·990 (0·987, 0·994)	< 0·001
All OSRs	Emergency EVAR	–0·00335	0·997 (0·994, 1·000)	0·033
	Emergency OSR	–0·00615	0·994 (0·993, 0·995)	< 0·001
	Elective EVAR	–0·00371	0·996 (0·992, 1·000)	0·069
	Elective OSR	–0·00666	0·993 (0·991, 0·995)	< 0·001
All EVARs	Emergency EVAR	–0·00056	0·999 (0·998, 1·001)	0·495
	Emergency OSR	–0·00260	0·997 (0·996, 0·998)	< 0·001
	Elective EVAR	0·00004	1·00 (0·998, 1·002)	0·971
	Elective OSR	–0·00192	0·998 (0·996, 1·000)	0·060

Values in parentheses are 95 per cent confidence intervals. EVAR, endovascular aneurysm repair; OSR, open surgical repair.

## Discussion

The data presented show that annual all‐procedure volume is predictive of outcomes and suggest that it is an appropriate overall measure of volume. OSR‐specific volume had a stronger influence on the volume effect than EVAR‐specific volume. This is an important finding, and relevant to clinicians and policymakers when planning future reconfiguration of AAA services. This is particularly important at a time when elective OSR is in decline and OSR remains the main method of repair for ruptured AAA^23^.

In agreement with previous studies in the UK^4^^,^^5^ and USA^17^^,^^27^, in the present study increased annual volumes were associated with significant reductions in in‐hospital mortality following OSR. The effect appeared to extend beyond the currently suggested threshold of 60 repairs per year to volumes above 100 repairs per year. However, there was no statistically significant relationship between volume and in‐hospital mortality after EVAR, in contrast to previous findings^5^^,^^15^^,^^17^. A UK study^5^ analysed HES data between 2005 and 2007, and found a statistically significant relationship between volume and in‐hospital mortality following EVAR. In an analysis^17^ of routine US data (Medicare) between 2001 and 2008, hospital volume was minimally associated with in‐hospital mortality after EVAR, whereas no such association was observed for surgeon volume.

The present study also evaluated whether there is a volume threshold to guide service reconfiguration. Current guidance from the service specification for vascular services^22^ and from the most recent version of a document from the Vascular Society of Great Britain and Ireland^28^ on the provision of vascular services suggests that centres should perform a minimum of 60 AAA repairs per year. The evidence presented here suggests that there would continue to be improvements in outcome if this threshold were increased to above 100 repairs per year. This may be related to the increased proportion of patients treated by EVAR, which may limit experience in OSR. These findings are important and could help in updating national and international guidelines for service reconfiguration^20^^,^^21^^,^^28^.

For other short‐term outcomes – duration of hospital stay and 30‐day readmissions – there was not enough evidence for significant volume–outcome relationships, although volume seemed to be associated with a slightly shorter stay for elective procedures if the volume of repairs exceeded about 100 per year. Another finding is the statistically significant relationship between volume and duration of critical care in admissions after emergency OSR; higher volume was associated with longer critical care stay. This could be related to the lower mortality observed in higher‐volume centres, where more patients survived at the expense of greater critical care requirements. Such a relationship between volume and critical care was not observed in other case‐mix groups. The findings regarding overall duration of hospital stay and critical care use may have implications in relation to service reconfiguration and resource constraints.

This study used improved methods for analysing administrative data for patients treated for AAA in the National Health Service (NHS) in England. The improved methods increased the validity of case identification and classification, as well as identification of sites. The EVAR procedures in this study were identified more completely than those in previous smaller studies. The study time frame covered a period of vascular services' reconfiguration in England that was driven by previous volume–outcome studies using the NHS administrative data set. The range of short‐term outcomes examined was also extended to include duration of hospital stay, 30‐day readmission and need for critical care.

Case‐mix adjustment was made for possible factors that could influence outcomes, including age, sex, year of data, deprivation index, weekend admission, mode of admission, type of procedure, and co‐morbidities evaluated using a modified version of the Charlson co‐morbidity categories^24^^,^^26^. Despite these efforts, there may be other important factors that were not included in the risk adjustment owing to the limitations of HES data. In particular, HES data do not include anatomical information and there may be aspects of patient selection that are not available for case‐mix adjustment. Higher‐volume centres tend to have higher EVAR rates^23^ and may be selecting patients with more complex anatomy for EVAR, and emergency data do not take account of selection that may take place in turning down patients for emergency procedures^24^. Although no relationship between volume and EVAR outcomes was observed, there may be competing effects as higher‐volume centres may be undertaking more complex EVAR procedures, a trend suggested by the recent National Vascular Registry report^23^. The recent increases in complex EVAR procedures, and differences in definitions between the National Vascular Registry and HES, require further investigation to understand the changes in patient selection and tertiary referrals, and the effect on cost and longer‐term outcomes. Emergency procedures (and elective ones to an extent) can also be confounded by turndown rates; only the effect on operated patients was evaluated here, and there is evidence of turndown rates being related to overall volumes and other factors, such as sex, with higher‐volume centres turning down fewer patients^24^.

This study only examined the relationships between volume and short‐term outcomes for patients who received repairs in hospitals; little is known about the impact of merging vascular centres and the effect that this has on long‐term outcomes, practice and patient selection. Although higher‐volume centres tend to have higher rates of EVAR, which are, *per se*, associated with lower inpatient mortality, critical care use and duration of hospital stay, economic modelling suggests that the reduced initial resource use and health benefits of this may be outweighed by higher overall costs and poorer long‐term outcomes^29^. Future research should investigate these relationships further and examine factors beyond volume to improve the quality of AAA surgical services.

## Supplementary Material

znaa179_Supplementary_Data

## References

[1] Phillips P , Poku E, Essat M, Woods HB, Goka EA, Kaltenthaler EC *et al*. Procedure volume and the association with short‐term mortality following abdominal aortic aneurysm repair in European populations: a systematic review. Eur J Vasc Endovasc Surg 2017;53:77–88. 27856168 10.1016/j.ejvs.2016.10.007

[2] Hentschker C , Mennicken R. The volume‐outcome relationship and minimum volume standards – empirical evidence for Germany. Health Econ 2015;24:644–658. 24700615 10.1002/hec.3051

[3] Trenner M , Haller B, Sollner H, Stork M, Umscheid T, Niedermeier H *et al*. Twelve years of the quality assurance registry on ruptured and non‐ruptured abdominal aortic aneurysms of the German Vascular Society (DGG). Part 3: predictors of the perioperative outcome with focus on annual caseload. Gefasschirurgie 2015;20:32–44.

[4] Holt PJ , Poloniecki JD, Loftus IM, Michaels JA, Thompson MM. Epidemiological study of the relationship between volume and outcome after abdominal aortic aneurysm surgery in the UK from 2000 to 2005. Br J Surg 2007;94:441–448. 17385180 10.1002/bjs.5725

[5] Holt PJ , Poloniecki JD, Khalid U, Hinchliffe RJ, Loftus IM, Thompson MM. Effect of endovascular aneurysm repair on the volume–outcome relationship in aneurysm repair. Circ Cardiovasc Qual Outcomes 2009;2:624–632. 20031901 10.1161/CIRCOUTCOMES.109.848465

[6] Holt PJ , Karthikesalingam A, Hofman D, Poloniecki JD, Hinchliffe RJ, Loftus IM *et al*. Provider volume and long‐term outcome after elective abdominal aortic aneurysm repair. Br J Surg 2012;99:666–672. 22344599 10.1002/bjs.8696

[7] Hafez H . National Vascular Database analysis: the relationship between AAA repair volume and outcome. Br J Surg 2012;99:4.22441847

[8] Eckstein HH , Bruckner T, Heider P, Wolf O, Hanke M, Niedermeier HP *et al*. The relationship between volume and outcome following elective open repair of abdominal aortic aneurysms (AAA) in 131 German hospitals. Eur J Vasc Endovasc Surg 2007;34:260–266. 17601754 10.1016/j.ejvs.2007.05.006

[9] Holt PJ , Karthikesalingam A, Poloniecki JD, Hinchliffe RJ, Loftus IM, Thompson MM. Propensity scored analysis of outcomes after ruptured abdominal aortic aneurysm. Br J Surg 2010;97:496–503. 20155793 10.1002/bjs.6911

[10] IMPROVE trial investigators; Powell JT, Hinchliffe RJ, Thompson MM, Sweeting MJ, Ashleigh R, Bell R*et al*. Observations from the IMPROVE trial concerning the clinical care of patients with ruptured abdominal aortic aneurysm. Br J Surg 2014;101:216–224. 24469620 10.1002/bjs.9410 PMC4164272

[11] Karthikesalingam A , Holt PJ, Vidal‐Diez A, Ozdemir BA, Poloniecki JD, Hinchliffe RJ *et al*. Mortality from ruptured abdominal aortic aneurysms: clinical lessons from a comparison of outcomes in England and the USA. Lancet 2014;383:963–969. 24629298 10.1016/S0140-6736(14)60109-4

[12] Ozdemir BA , Karthikesalingam A, Sinha S, Poloniecki JD, Vidal‐Diez A, Hinchliffe RJ *et al*. Association of hospital structures with mortality from ruptured abdominal aortic aneurysm. Br J Surg 2015;102:516–524. 25703735 10.1002/bjs.9759

[13] Jibawi A , Hanafy M, Guy A. Is there a minimum caseload that achieves acceptable operative mortality in abdominal aortic aneurysm operations? Eur J Vasc Endovasc Surg 2006;32:273–276. 16725357 10.1016/j.ejvs.2006.03.013

[14] Karthikesalingam A , Wanhainen A, Holt PJ, Vidal‐Diez A, Brownrigg JR, Shpitser I *et al*. Comparison of long‐term mortality after ruptured abdominal aortic aneurysm in England and Sweden. Br J Surg 2016;103:199–206. 26620854 10.1002/bjs.10049

[15] Karthikesalingam A , Holt PJ, Vidal‐Diez A, Bahia SS, Patterson BO, Hinchliffe RJ *et al*. The impact of endovascular aneurysm repair on mortality for elective abdominal aortic aneurysm repair in England and the United States. J Vasc Surg 2016;64:321–327. 27050198 10.1016/j.jvs.2016.01.057

[16] Trenner M , Kuehnl A, Salvermoser M, Reutersberg B, Geisbuesch S, Schmid V *et al*. Editor's choice –high annual hospital volume is associated with decreased in hospital mortality and complication rates following treatment of abdominal aortic aneurysms: secondary data analysis of the nationwide German DRG statistics from 2005 to 2013. Eur J Vasc Endovasc Surg 2018;55:185–194. 29289619 10.1016/j.ejvs.2017.11.016

[17] Zettervall SL , Schermerhorn ML, Soden PA, McCallum JC, Shean KE, Deery SE *et al*. The effect of surgeon and hospital volume on mortality after open and endovascular repair of abdominal aortic aneurysms. J Vasc Surg 2017;65:626–634. 27988158 10.1016/j.jvs.2016.09.036 PMC5329005

[18] Beck AW , Sedrakyan A, Mao J, Venermo M, Faizer R, Debus S *et al*. Variations in abdominal aortic aneurysm care: a report from the international consortium of vascular registries. Circulation 2016;134:1948–1958. 27784712 10.1161/CIRCULATIONAHA.116.024870 PMC5147037

[19] Scali ST , Beck AW, Sedrakyan A, Mao J, Venermo M, Faizer R *et al*. Hospital volume association with abdominal aortic aneurysm repair mortality: analysis of the International Consortium of Vascular Registries. Circulation 2019;140:1285–1287. 31589486 10.1161/CIRCULATIONAHA.119.042504

[20] Moll FL , Powell JT, Fraedrich G, Verzini F, Haulon S, Waltham M *et al*.; European Society for Vascular Surgery. Management of abdominal aortic aneurysms clinical practice guidelines of the European Society for Vascular Surgery. Eur J Vasc Endovasc Surg 2011;41:S1–S58. 21215940 10.1016/j.ejvs.2010.09.011

[21] Leapfrog Group. *Inpatient Surgery Report 2019*; 2019. https://www.leapfroggroup.org/inpatient‐surgery‐report‐2019 [accessed 9 October 2019].

[22] England NHS; Department of Health. Service Specifications No 1700004/S ‐ Specialised Vascular Services (Adults). NHS England: London, 2016.

[23] Waton S , Johal A, Heikkila K, Cromwell D, Boyle J, Miller F. National Vascular Registry: 2018 Annual Report. Royal College of Surgeons of England: London, 2018.

[24] Aber A , Tong TS, Chilcott J, Thokala P, Maheswaran R, Thomas SM *et al*. Sex differences in national rates of repair of emergency abdominal aortic aneurysm. Br J Surg 2019;106:82–89. 30395361 10.1002/bjs.11006

[25] Aber A , Tong T, Chilcott J, Maheswaran R, Thomas SM, Nawaz S *et al*. Outcomes of aortic aneurysm surgery in England: a nationwide cohort study using hospital admissions data from 2002 to 2015. BMC Health Serv Res 2019;19:988. 31870354 10.1186/s12913-019-4755-0 PMC6929362

[26] Armitage JN , van der Meulen JH. Identifying co‐morbidity in surgical patients using administrative data with the Royal College of Surgeons Charlson Score. Br J Surg 2010;97:772–781. 20306528 10.1002/bjs.6930

[27] Landon BE , O'Malley AJ, Giles K, Cotterill P, Schermerhorn ML. Volume–outcome relationships and abdominal aortic aneurysm repair. Circulation 2010;122:1290–1297. 20837892 10.1161/CIRCULATIONAHA.110.949172

[28] Vascular Society of Great Britain and Ireland. The Provision of Services for Patients with Vascular Disease 2018. Glasgow: Vascular Society of Great Britain and Ireland, 2018.

[29] National Institute for Health and Care Excellence (NICE). Abdominal Aortic Aneurysm: Diagnosis and Management. NICE guideline NG156; 2020. https://www.nice.org.uk/guidance/ng156 [accessed 19 May 2020]. 32407016

