# Supplementary material for: Volume–outcome relationships in open and endovascular repair of abdominal aortic aneurysm: administrative data 2006–2018
Source: Br J Surg. 2021 May 26;108(5):521–7. doi: 10.1002/bjs.11919 (PMC10364891; doi:10.1002/bjs.11919)
Supplement: znaa179_Supplementary_Data [file znaa179_supplementary_data.docx]

**BJS11919**

**Volume–outcome relationships in open and endovascular repair of abdominal aortic aneurysm: administrative data 2006–2018**

T. Tong, A. Aber, J. Chilcott, P. Thokala, S. J. Walters, R. Maheswaran, S. Nawaz, S. Thomas and J. Michaels

**Appendix S1** A comparison between AAA repair cases per provider identified from Hospital Episode Statistics (HES) data and the reported cases in the NVR report 2018

1. INTRODUCTION

Using a broad filter based health resource groups (HRG) codes, office of population census and surveys (OPCS) codes, the 10^th^ revision of International Classification of Diseases (ICD) codes, and treatment specialty codes; vascular related inpatient Hospital Episode Statistics (HES) data from the financial year 2006/2007 to 2017/2018 were acquired from NHS Digital. From this broad extract of vascular episodes, specific filters were then developed to identify and classify AA-related episodes into clinical sub-groups including infra-renal repairs versus complex repairs, ruptured repairs versus non-ruptured emergency repairs versus elective repairs, and Endovascular Aneurysm Repair (EVAR) versus Open Repair. More details of our methods to identify and classify AA-related episodes are referred to Aber et al. ^1^

The aim of this study is to further validate our methods by comparing the number of repairs cases per provider identified from HES data with the numbers reported in the National Vascular Registry (NVR) 2018-report ^2^.

1. METHODS

The NVR annual report (2018) was published online on their website (https://www.vsqip.org.uk/reports/2018-annual-report/). The main report (2018) presents the summary statistics of AAA repair cases for three groups of patients in the UK (including England, Wales, Scotland and Northern Ireland): elective infrarenal AAA repairs (cases registered between Jan 2017 and December 2017), elective complex AAA repairs (registered between Jan 2015 and December 2017), and ruptured AAA repairs (registered between Jan 2015 and December 2017). It should be noted that the reported number for elective infrarenal cases was for one year (2017) whereas the reported numbers for elective complex repair cases and ruptured repair cases were for three years combined (from 2015 to 2017). The reported numbers for each trust (defined by unique 3-character provider code) are provided in the appendices (available online).

On the other hand, our HES data only included cases in England. Thus, NVR cases in Scotland, Wales and Northern Ireland need to be excluded from the NVR estimates. HES data were structured by the financial years (from 1^st^ April to 31^st^ March). To identify cases from HES data that are comparable with the NVR data dates (i.e. between Jan 2017 and December 2017), the operation date in HES data was used. For example, cases with operation dates between 1^st^ Jan 2017 and 31^st^ December 2017 were selected to compare with the NVR reported elective infrarenal AAA repairs. Because a patient can have multiple admissions in HES data, there are two ways to define a case of AAA repair: only counting unique patients receiving AAA repair versus counting separate hospital admissions with AAA repair. Although majority of AAA patients only have one admission with AAA repair, some patients actually have multiple AAA repair admissions (note that we use Continuous Inpatient Stay to define an admission, hence, excluding hospital transfers as separate admissions). In this validation study, we count unique patients in HES to compare with the NVR reported numbers. After an initial investigation comparing the 3-character provider codes between the NVR (2018) and HES, we found two instances that need correction/adjustment. First, the NVR provider code for Manchester University NHS Foundation Trust was R0A whereas it was RW3 in HES and Reference Cost data. The correction was applied on the NVR data to change R0A to RW3. Second, the Wythenshawe hospital in Manchester was considered under the Manchester University NHS Foundation Trust, however the 3-character provider code in HES data was RM2. The correction was then applied on HES data to change RM2 to RW3 so that the estimates of Wythenshawe hospital could be counted for the Manchester University NHS Foundation Trust.

The HES estimates for elective infra-renal repairs, elective complex repairs, and ruptured repairs were compared with the NVR estimates as follows. First, the estimates for each sub-group were compared and discussed for England (as a whole) and for each trust. Then, the total combined estimates (including all three sub-groups) were compared to see the overall level of agreement between HES and NVR. To maintain anonymization, some trusts with less 5 cases were reported with “<5” in the NVR. For these trusts, if their numbers are also less than 5 cases in HES data, we assumed a perfect match between HES and NVR; if their numbers are not less than 5 cases in HES data, we assumed the NVR number was 4 to make a comparison.

1. RESULTS

- 1. **Compare elective infra-renal repairs between HES and NVR**

The NVR reported 4,208 elective infra-renal AAA repairs between Jan 2017 and December 2017. 1338 (32%) were open repair and 2870 (68%) were EVAR. If excluding Scotland, Wales and Northern Ireland (to be comparable with HES data), between Jan 2017 and December 2017, the NVR estimated 3622 elective infra-renal AAA repairs, 1130 (31%) were open repair and 2492 (69%) were elective EVAR

From HES data (England), our algorithm identified 5177 infra-renal patients with AAA repairs between 1^st^ Jan 2017 and 31^st^ December 2017 (based on operation date).3875 (75%) were elective. 1187 (31% of elective cases) were elective open repair. 2688 (69%) were elective EVAR.

Fig. S1 shows the trust-specific comparison for the number of elective infra-renal repair cases between NVR and HES. The straight line represents the perfect match between HES and NVR (the number from HES is the same as the number from NVR). The points above the line represent trusts where the number from NVR is higher than the number from HES. The points below the line represents trusts where the number from NVR is lower than the number from HES. There are more points below the line (especially towards the higher numbers) suggesting HES picking up more cases than NVR. We can see a strong positive correlation between the numbers from NVR and the numbers from HES (Person Product-Moment Correlation Coefficient = 0.95).

Fig. S1: trust-specific elective infra-renal cases between Jan 2017 and December 2017 (NVR vs HES)

- 1. **Compare elective complex AAA repairs between HES and NVR**

The NVR reported 2303 elective complex AAA repairs between Jan 2015 and December 2017. 229 (10%) were open repair and 2074 (90%) were EVAR. If excluding Scotland, Wales and Northern Ireland (to be comparable with HES data), between Jan 2015 and December 2017, the NVR estimated 2147 elective complex AAA repairs, 193 (9%) were elective open complex repair and 1954 (91%) were elective complex EVAR

From HES data (England only), we identified 2727 patients with complex AAA repairs between 1^st^ Jan 2015 and 31^st^ December 2017 (based on operation date). 1723 (63%) were elective. 506 (29% of elective cases) were elective open repair. 1217 (71%) were elective EVAR.

Fig. S2 shows the trust-specific comparison for the number of elective complex repair cases between NVR and HES. There are more points above the (x=y) line (especially towards the higher numbers) suggesting NVR picking up more complex repair cases than HES. There is still a strong positive correlation between the numbers from NVR and the numbers from HES (Person Product-Moment Correlation Coefficient = 0.93), however, it is not as strong as elective infra-renal cases. Compare to elective infra-renal cases, elective complex repairs have more trusts with substantially wide differences between the number from NVR and the number from HES.

Fig. S2: trust-specific elective complex cases between Jan 2015 and December 2017 (NVR vs HES)

- 1. **Compare ruptured AAA repairs between HES and NVR**

The NVR (2018) reported 2681 ruptured AAA repairs between Jan 2015 and December 2017; about 30% were EVAR repair. If excluding Scotland, Wales and Northern Ireland (to be comparable with HES data), between Jan 2015 and December 2017, the NVR estimated 2281 ruptured repairs with 32% were ruptured EVAR repair (735 cases).

From HES data (England only), we identified 2757 patients with ruptured AAA repairs between 1^st^ Jan 2015 and 31^st^ December 2017 (based on operation date). 1876 (68% of ruptured cases) were ruptured open repair. 881 (32% of ruptured cases) were ruptured EVAR.

*Fig. S3* shows the trust-specific comparison for the number of elective complex repair cases between NVR and HES. There are more points below the (x=y) line suggesting HES picking up more ruptured repair cases than NVR. There is still a strong positive correlation between the numbers from NVR and the numbers from HES (Person Product-Moment Correlation Coefficient = 0.85), however, it is not as strong as elective infra-renal cases. There are more trusts with substantially wide differences between the number from NVR and the number from HES.

Fig. S3: trust-specific ruptured repair cases between Jan 2015 and December 2017 (NVR vs HES)

- 1. **Compare total AAA repairs between HES and NVR**

Combining all cases for elective infra-renal repairs (between Jan 2017 and December 2017), elective complex repairs (between Jan 2015 and December 2017), and ruptured repairs (between Jan 2015 and December 2017), the NVR (2018) reported for England a total of 8050 repair cases, 5181 (64%) were EVAR and 2869 (36%) were Open Repair. From HES data (England only), the equivalent total repair cases was 8355 cases, 4786 (57%) were EVAR and 3569 (43%) were Open Repair. This makes a case ascertainment (NVR vs HES) of 96% for the NVR.

Fig. S4 shows the trust-specific comparison for the total number of repair cases between NVR and HES. There is still a strong positive correlation between the numbers from NVR and the numbers from HES (Person Product-Moment Correlation Coefficient = 0.93).

Fig. S4: trust-specific total cases NVR versus HES

1. CONCLUSIONS

This validation study shows that our algorithms to identify and classify cases of AAA repairs from HES data performed well. The results fit with what we expected given the known limitations of HES data ^3, 4^. There is good matching/agreement between HES and NVR. The numbers from HES and the numbers from NVR are strongly correlated. The algorithms seemed to perform better in identifying infra-renal cases than complex cases and ruptured cases.

REFERENCES

1. Aber A, Tong T, Chilcott J, Maheswaran R, Thomas SM, Nawaz S, Michaels J. Outcomes of aortic aneurysm surgery in England: a nationwide cohort study using hospital admissions data from 2002 to 2015. *BMC Health Serv Res* 2019;**19**(1): 988.

2. Waton S, Johal A, Heikkila K, Cromwell D, Boyle J, Miller F. National Vascular Registry: 2018 Annual Report. In: The Royal College of Surgeons of England; 2018.

3. Herbert A, Wijlaars L, Zylbersztejn A, Cromwell D, Hardelid P. Data Resource Profile: Hospital Episode Statistics Admitted Patient Care (HES APC). *International Journal of Epidemiology* 2017;**46**(4): 1093-1093i.

4. Bottle A, Gaudoin R, Goudie R, Jones S, Aylin P. Can valid and practical risk-prediction or casemix adjustment models, including adjustment for comorbidity, be generated from English hospital administrative data (Hospital Episode Statistics)? A national observational study. *NIHR Journals Library* 2014.

**Appendix S2** A multi-level modelling approach to analyse the volume–outcome relationship in repair of abdominal aortic aneurysm from hospital episode statistics (HES) data

1. INTRODUCTION

In the main text of the paper, a single level multivariate model was employed to investigate the volume-outcome relationship in repair of Abdominal Aortic Aneurysm (AAA) from HES data. The aim of this study is to investigate the volume-outcome relationship using a multi-level modelling approach.

1. METHODS

After an initial investigation of the data with a multi-level modelling approach, two levels were determined to be appropriate given the data structure: level one is patient level and level two is defined by a combination of hospital identification and HESyear. HESyear was incorporated in level two because year-specific volume associated with a hospital was the main measure of volume (as discussed in the main text of our paper). This would account for the historical reconfiguration of vascular services in the data.

Level-1 explanatory variables include age, gender, Index of Multiple Deprivation (IMD), comorbidities, ruptured aneurysm and weekend admission (for emergency cases). Level-2 explanatory variable is year-specific hospital volume. Continuous variables (age, IMD, and volume) were standardised by subtracting the mean and dividing by the standard deviation. Two-level (random intercept) logistic regression models were employed to investigate the relationship between year-specific volume and in-hospital death. Models were fitted separately for four groups: emergency EVAR, emergency Open Repair, elective EVAR, and elective Open Repair. For each group, first, a null or empty two-level model was fitted to understand the data without explanatory variables. Then, two-level models were explanatory variables were fitted to understand the relationship between year-specific volume and in-hospital death. Only random intercept models were investigated due to the explanatory nature of the study and time and resources constraints.

The null or empty two-level model only includes an intercept and year-specific-hospital effects. This is illustrated in the equation below:

$$Logit\left( P\left( Y_{ij}=1 \right) \right)=\beta_{0}+u_{0j}$$

The intercept $\beta_{0}$ is shared by all year-specific hospitals while the random effect $u_{0j}$ is specific to year-specific-hospital j. The random effect is assumed to follow a normal distribution with variance ${}_{u0}^{2}$.

The two-level model with explanatory variables at level 1 and level 2 is described below.

$$Logit\left( P\left( Y_{ij}=1 \right) \right)=\beta_{0}+ \beta_{1}{Age}_{ij}+ \beta_{2}{Male}_{ij}+ \beta_{3}{IMD}_{ij}+ \beta_{4}{Comorbidities}_{ij}+\beta_{5}{Ruptured}_{ij}+ \beta_{6}{Weekend}_{ij}+ \beta_{7}{Volume}_{j}+ u_{0j}$$

All data manipulation was performed in R (Version 3.4.1) (R foundation, Vienna, Austria). All models were fitted using the R package ‘lme4’ (Bates, Mächler et al., 2015).

1. RESULTS
   1. **Volume outcome relationship for emergency EVAR**

*The null two-level model:*

| **Methods:**   - Generalized linear mixed model fit by maximum likelihood (Laplace Approximation) ['glmerMod'] - Family: binomial (logit) - Number of observations (patients): 5719 - Number of groups (siteyear): 832 | | | | | |
| --- | --- | --- | --- | --- | --- |
| **Random effects:** | | | | | |
|  | *Groups* | *Name* | *Variance* | *Std.Dev* |  |
|  | siteyear | (Intercept) | 5.73E-12 | 2.39E-06 |  |
| **Fixed effects** | | | | | |
|  | *Name* | *Estimate* | *Std.Error* | *Z value* | *Pr(>\|z\|)* |
|  | (Intercept) | -1.93298 | 0.03979 | -48.58 | <2e-16 |
| **Note:** boundary (singular) fit | | | | | |

The log-odds of in-hospital death in an ‘average’ year-specific hospital (one with $u_{0j}=0$ ) is estimated as ${}_{0}$ (hat) = - 1.93 (Mortality = 12.64% ). The intercept for year-specific hospital j is -1.93122 + $u_{0j}$, where the variance (between year-specific hospitals) of $u_{0j}$ is estimated as ${}_{u0}^{2}$(hat) = 5.73E-12 (~ zero). The fitted mixed model is singular: the variance for groups is close to zero.

The likelihood ratio statistic for testing the null hypothesis, that ${}_{u0}^{2}=0$, can be calculated by comparing the two-level model, with the corresponding single-level model without the level 2 random effects. The test statistic is ~ 0 with 1 degree of freedom so there is no evidence that the between year-specific hospital variance is different from zero.

*The two-level model with explanatory variables:*

| **Methods:**   - Generalized linear mixed model fit by maximum likelihood (Laplace Approximation) ['glmerMod'] - Family: binomial (logit) - Number of observations (patients): 5617 - Number of groups (siteyear): 828 | | | | | |
| --- | --- | --- | --- | --- | --- |
| **Random effects:** | | | | | |
|  | *Groups* | *Name* | *Variance* | *Std.Dev* |  |
|  | siteyear | (Intercept) | 1.167E-22 | 1.08E-11 |  |
| **Fixed effects** | | | | | |
| *Name* | | *Estimate* | *Std.Error* | *Z value* | *Pr(>\|z\|)* |
| Intercept | | -2.79 | 0.13 | -21.47 | 0.000 |
| Standardised Age | | 0.34 | 0.05 | 6.99 | 0.000 |
| Male (vs Female) | | -0.26 | 0.11 | -2.38 | 0.017 |
| Ruptured AAA | | 1.75 | 0.09 | 18.83 | 0.000 |
| Admitted in weekend | | 0.20 | 0.10 | 1.93 | 0.054 |
| Standardised Index of Multiple Deprivation (2004) | | 0.05 | 0.04 | 1.06 | 0.288 |
| Coronary Artery Disease | | -0.02 | 0.11 | -0.20 | 0.843 |
| Heart Failure | | 0.05 | 0.16 | 0.31 | 0.753 |
| COPD | | 0.28 | 0.09 | 3.07 | 0.002 |
| Diabetes | | -0.12 | 0.13 | -0.91 | 0.360 |
| Renal Disease | | 0.31 | 0.15 | 2.15 | 0.031 |
| Cancer | | -0.19 | 0.16 | -1.19 | 0.234 |
| Moderate or Severe Liver Disease | | 1.87 | 0.46 | 4.09 | 0.000 |
| Dyslipidemia | | -0.22 | 0.10 | -2.11 | 0.035 |
| Standardised Annual AAA Volume | | -0.07 | 0.04 | -1.56 | 0.119 |
| **Note:** boundary (singular) fit | | | | | |

After adjustment for age, gender, Index of Multiple Deprivation (IMD), comorbidities, ruptured aneurysm and weekend admission, the between year-specific hospital variance is still close to zero (singular fit). There seems to be a negative relationship between volume and in-hospital death for emergency EVAR cases (higher volume associates with lower mortality), however, it is not significant (P value = 0.12).

- 1. **Volume outcome relationship for emergency Open Repair**

*The null two-level model:*

| **Methods:**   - Generalized linear mixed model fit by maximum likelihood (Laplace Approximation) ['glmerMod'] - Family: binomial (logit) - Number of observations (patients): 15,953 - Number of groups (siteyear): 1,359 | | | | | |
| --- | --- | --- | --- | --- | --- |
| **Random effects:** | | | | | |
|  | *Groups* | *Name* | *Variance* | *Std.Dev* |  |
|  | siteyear | (Intercept) | 0.08982 | 0.2997 |  |
| **Fixed effects** | | | | | |
|  | *Name* | *Estimate* | *Std.Error* | *Z value* | *Pr(>\|z\|)* |
|  | (Intercept) | -0.77798 | 0.0199 | -39.09 | <2e-16 |

The log-odds of in-hospital death in an ‘average’ year-specific hospital (one with $u_{0j}=0$ ) is estimated as ${}_{0}$ (hat) = - 0.78 (Mortality = 31.48% ). The intercept for year-specific hospital j is -0.78 + $u_{0j}$, where the variance (between year-specific hospitals) of $u_{0j}$ is estimated as ${}_{u0}^{2}$(hat) = 0.09.

The likelihood ratio statistic for testing the null hypothesis, that ${}_{u0}^{2}=0$, can be calculated by comparing the two-level model, with the corresponding single-level model without the level 2 random effects. The test statistic is 44.3 with 1 degree of freedom so there is evidence that the between year-specific hospital variance is non-zero.

The estimates of year-specific hospital effects or residuals, $u_{0j}$ (hat) obtained from the null model can be examined in a caterpillar plot where the hospital effects shown in rank order together with 95% CI. This is illustrated in Fig. S5 below.

Fig. S5: The estimated year-specific hospital effects for emergency Open Repair


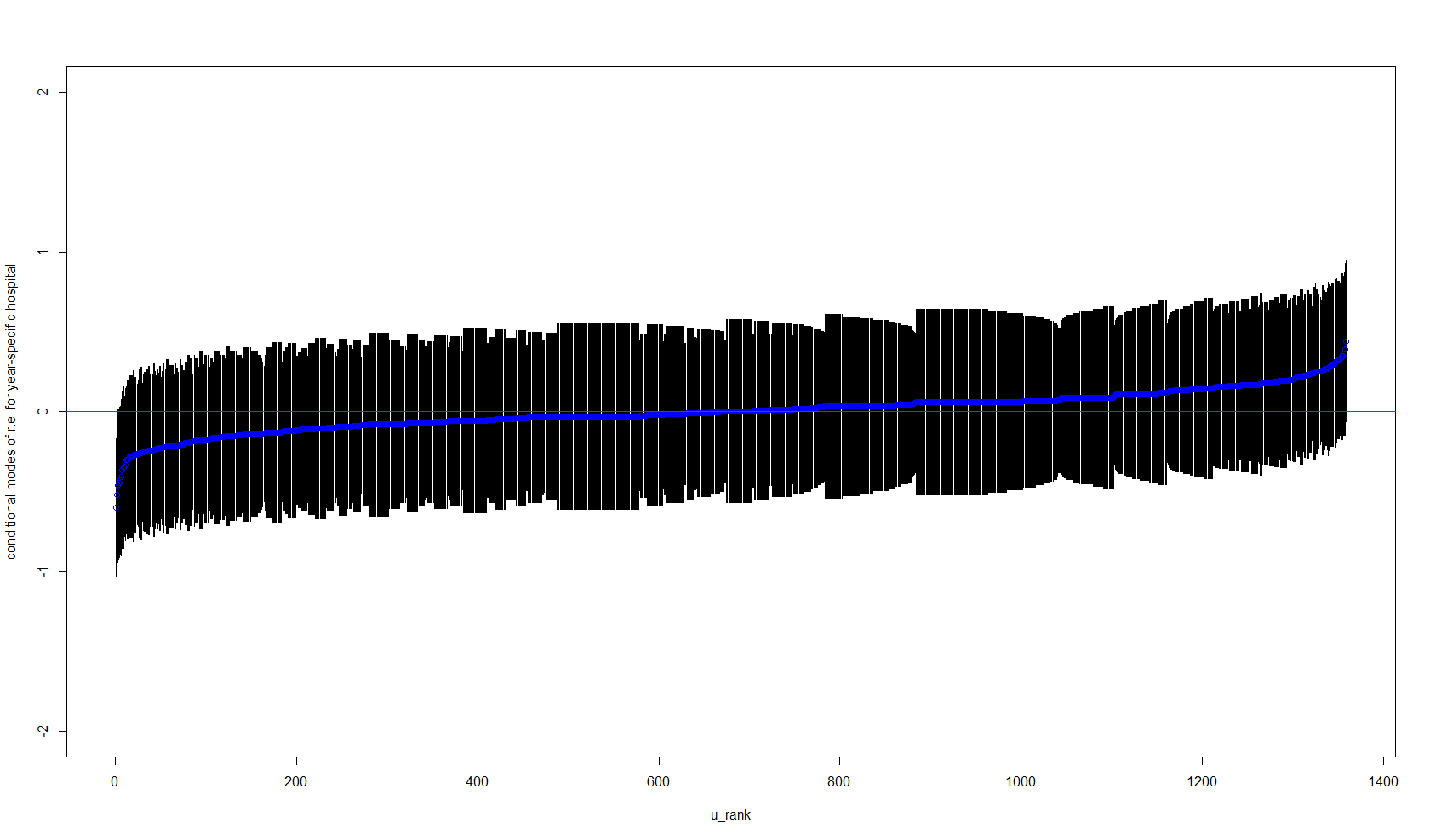


The plot shows the estimated residuals for all 1,359 different year-specific hospitals in the sample. All year-specific hospitals have their 95% confidence interval overlap the horizontal line at zero, indicating a consistent in-hospital mortality rate across year-specific hospitals (around the national average).

*The two-level model with explanatory variables:*

| **Methods:**   - Generalized linear mixed model fit by maximum likelihood (Laplace Approximation) ['glmerMod'] - Family: binomial (logit) - Number of observations (patients): 15,680 - Number of groups (siteyear): 1,355 | | | | | |
| --- | --- | --- | --- | --- | --- |
| **Random effects:** | | | | | |
|  | *Groups* | *Name* | *Variance* | *Std.Dev* |  |
|  | siteyear | (Intercept) | 0.06887 | 0.2624 |  |
| **Fixed effects** | | | | | |
| *Name* | | *Estimate* | *Std.Error* | *Z value* | *Pr(>\|z\|)* |
| Intercept | | -1.90 | 0.06 | -30.79 | 0.000 |
| Standardised Age | | 0.56 | 0.02 | 24.05 | 0.000 |
| Male (vs Female) | | -0.16 | 0.05 | -3.33 | 0.001 |
| Ruptured AAA | | 1.35 | 0.05 | 28.80 | 0.000 |
| Admitted in weekend | | 0.16 | 0.04 | 3.55 | 0.000 |
| Standardised Index of Multiple Deprivation (2004) | | 0.07 | 0.02 | 3.86 | 0.000 |
| Coronary Artery Disease | | 0.19 | 0.06 | 3.43 | 0.001 |
| Heart Failure | | 0.18 | 0.10 | 1.75 | 0.081 |
| COPD | | 0.25 | 0.05 | 5.49 | 0.000 |
| Diabetes | | 0.14 | 0.06 | 2.45 | 0.014 |
| Renal Disease | | 0.57 | 0.10 | 5.96 | 0.000 |
| Cancer | | 0.08 | 0.08 | 0.92 | 0.356 |
| Moderate or Severe Liver Disease | | 3.08 | 0.24 | 13.07 | 0.000 |
| Dyslipidemia | | -0.15 | 0.05 | -3.13 | 0.002 |
| Standardised Annual AAA Volume | | -0.16 | 0.02 | -7.41 | 0.000 |

Adding explanatory variables reduced the estimate of the between year-specific hospital variance (from 0.08982 to 0.06887). There is a negative and significant (P-value < 0.001) relationship between volume and in-hospital death for emergency Open Repair cases (higher volume associates with lower mortality).

- 1. **Volume outcome relationship for elective EVAR**

*The null two-level model:*

| **Methods:**   - Generalized linear mixed model fit by maximum likelihood (Laplace Approximation) ['glmerMod'] - Family: binomial (logit) - Number of observations (patients): 28,656 - Number of groups (siteyear): 970 | | | | | |
| --- | --- | --- | --- | --- | --- |
| **Random effects:** | | | | | |
|  | *Groups* | *Name* | *Variance* | *Std.Dev* |  |
|  | siteyear | (Intercept) | 0.5073 | 0.7122 |  |
| **Fixed effects** | | | | | |
|  | *Name* | *Estimate* | *Std.Error* | *Z value* | *Pr(>\|z\|)* |
|  | (Intercept) | (Intercept) | -4.58 | 0.0887 | <0.001 |

The log-odds of in-hospital death in an ‘average’ year-specific hospital (one with $u_{0j}=0$ ) is estimated as ${}_{0}$ (hat) = - 4.58 (Mortality = 1.01% ). The intercept for year-specific hospital j is -4.58 + $u_{0j}$, where the variance (between year-specific hospitals) of $u_{0j}$ is estimated as ${}_{u0}^{2}$(hat) = 0.5.

The likelihood ratio statistic for testing the null hypothesis, that ${}_{u0}^{2}=0$, can be calculated by comparing the two-level model, with the corresponding single-level model without the level 2 random effects. The test statistic is 13.78 with 1 degree of freedom so there is evidence that the between year-specific hospital variance is non-zero.

The estimates of year-specific hospital effects or residuals, $u_{0j}$ (hat) obtained from the null model can be examined in a caterpillar plot where the hospital effects shown in rank order together with 95% CI. This is illustrated in Fig. S6 below.

Fig. S6 The estimated year-specific hospital effects for elective EVAR


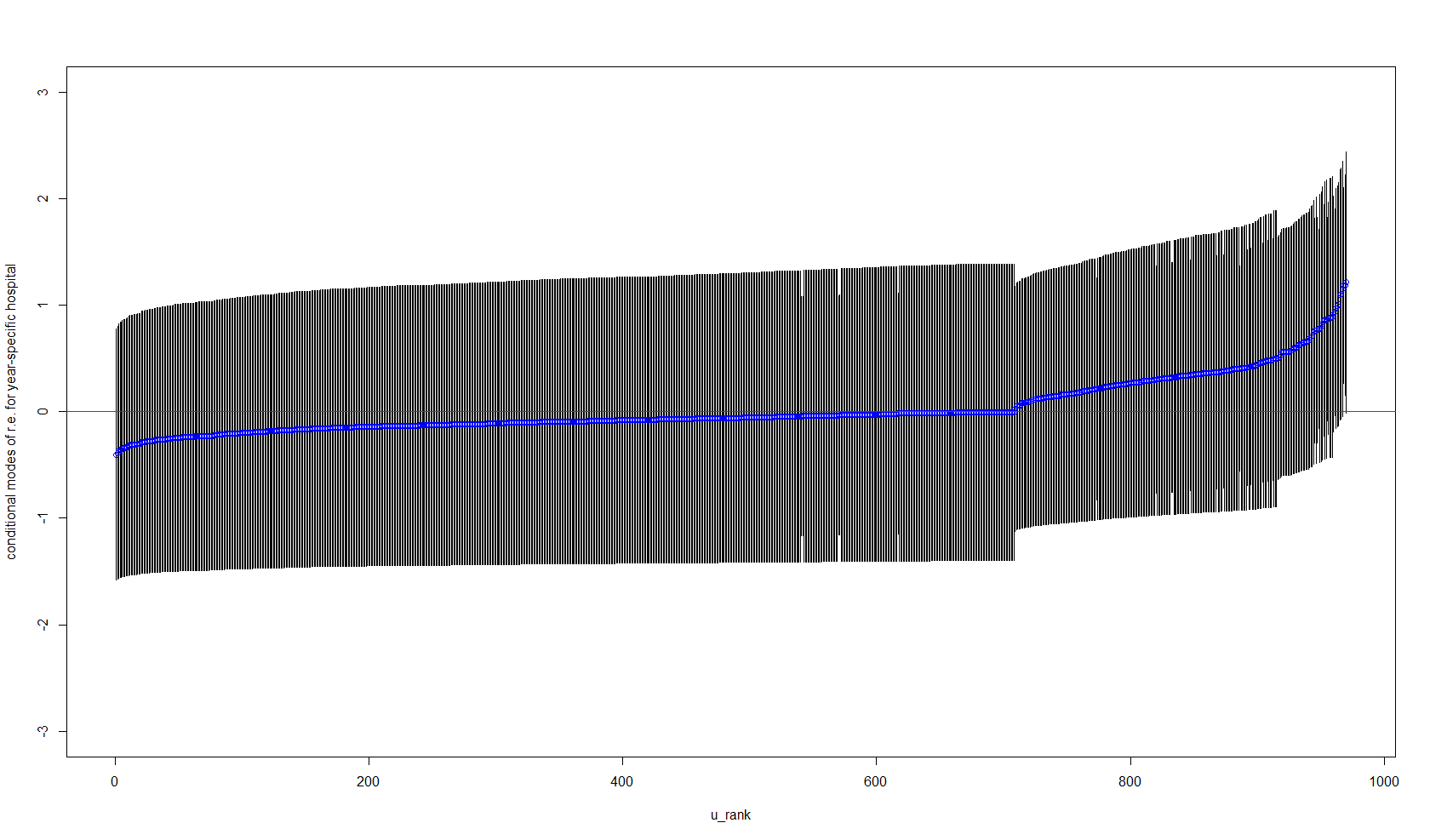


The plot shows the estimated residuals for all 970 different year-specific hospitals in the sample. All year-specific hospitals have their 95% confidence interval overlap the horizontal line at zero, indicating a consistent in-hospital mortality rate across year-specific hospitals (around the national average).

*The two-level model with explanatory variables:*

| **Methods:**   - Generalized linear mixed model fit by maximum likelihood (Laplace Approximation) ['glmerMod'] - Family: binomial (logit) - Number of observations (patients): 28,204 - Number of groups (siteyear): 970 | | | | | |
| --- | --- | --- | --- | --- | --- |
| **Random effects:** | | | | | |
|  | *Groups* | *Name* | *Variance* | *Std.Dev* |  |
|  | siteyear | (Intercept) | 0.4844 | 0.696 |  |
| **Fixed effects** | | | | | |
| *Name* | | *Estimate* | *Std.Error* | *Z value* | *Pr(>\|z\|)* |
| Intercept | | -4.40 | 0.16 | -27.13 | 0.000 |
| Standardised Age | | 0.25 | 0.06 | 4.21 | 0.000 |
| Male (vs Female) | | -0.66 | 0.14 | -4.75 | 0.000 |
| Standardised Index of Multiple Deprivation (2004) | | 0.09 | 0.05 | 1.69 | 0.091 |
| Coronary Artery Disease | | 0.49 | 0.12 | 4.12 | 0.000 |
| Heart Failure | | 0.13 | 0.19 | 0.72 | 0.474 |
| COPD | | 0.36 | 0.12 | 3.12 | 0.002 |
| Diabetes | | 0.03 | 0.14 | 0.19 | 0.849 |
| Renal Disease | | 0.42 | 0.16 | 2.63 | 0.009 |
| Cancer | | 0.06 | 0.16 | 0.37 | 0.715 |
| Moderate or Severe Liver Disease | | 2.69 | 0.32 | 8.48 | 0.000 |
| Dyslipidemia | | -0.20 | 0.12 | -1.62 | 0.104 |
| Standardised Annual AAA Volume | | -0.10 | 0.06 | -1.52 | 0.130 |

Adding explanatory variables reduced the estimate of the between year-specific hospital variance (from 0.5073 to 0.4844). There seems to be a negative relationship between volume and in-hospital death for elective EVAR cases (higher volume associates with lower mortality), however, it is not significant (P value = 0.13).

- 1. **Volume outcome relationship for elective Open Repair**

*The null two-level model:*

| **Methods:**   - Generalized linear mixed model fit by maximum likelihood (Laplace Approximation) ['glmerMod'] - Family: binomial (logit) - Number of observations (patients): 21,694 - Number of groups (siteyear): 1,294 | | | | | |
| --- | --- | --- | --- | --- | --- |
| **Random effects:** | | | | | |
|  | *Groups* | *Name* | *Variance* | *Std.Dev* |  |
|  | siteyear | (Intercept) | 0.2988 | 0.5466 |  |
| **Fixed effects** | | | | | |
|  | *Name* | *Estimate* | *Std.Error* | *Z value* | *Pr(>\|z\|)* |
|  | (Intercept) | -3.00238 | 0.0415 | -72.34 | <2e-16 |

The log-odds of in-hospital death in an ‘average’ year-specific hospital (one with $u_{0j}=0$ ) is estimated as ${}_{0}$ (hat) = - 3.00 (In-hospital Mortality = 4.73% ). The intercept for year-specific hospital j is -3.00 + $u_{0j}$, where the variance (between year-specific hospitals) of $u_{0j}$ is estimated as ${}_{u0}^{2}$(hat) = 0.2988.

The likelihood ratio statistic for testing the null hypothesis, that ${}_{u0}^{2}=0$, can be calculated by comparing the two-level model, with the corresponding single-level model without the level 2 random effects. The test statistic is 36.28 with 1 degree of freedom so there is evidence that the between year-specific hospital variance is non-zero.

The estimates of year-specific hospital effects or residuals, $u_{0j}$ (hat) obtained from the null model can be examined in a caterpillar plot where the hospital effects shown in rank order together with 95% CI. This is illustrated in Fig. S7 below.

Fig. S7 The estimated year-specific hospital effects for elective Open Repair


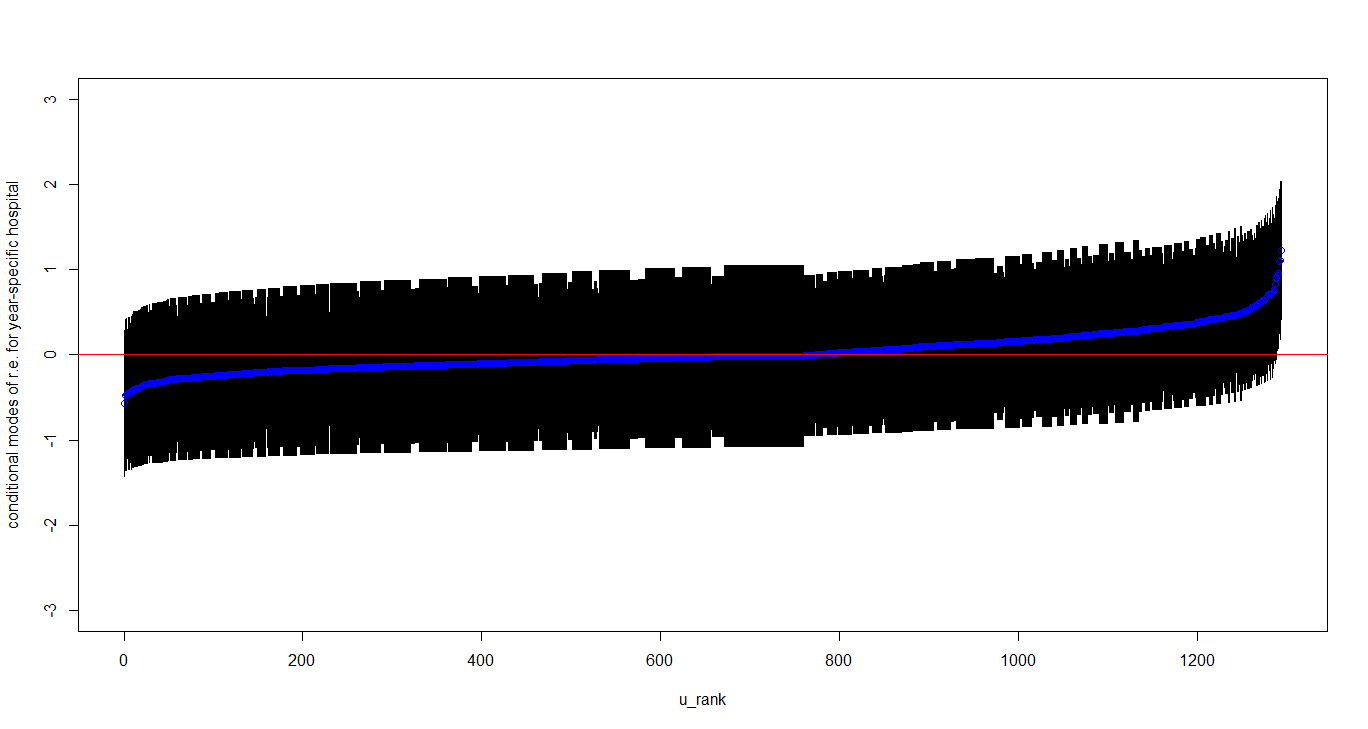


The plot shows the estimated residuals for all 1,294 different year-specific hospitals in the sample. All year-specific hospitals have their 95% confidence interval overlap the horizontal line at zero, indicating a consistent in-hospital mortality rate across year-specific hospitals (around the national average).

*The two-level model with explanatory variables:*

| **Methods:**   - Generalized linear mixed model fit by maximum likelihood (Laplace Approximation) ['glmerMod'] - Family: binomial (logit) - Number of observations (patients): 21,422 - Number of groups (siteyear): 1,292 | | | | | |
| --- | --- | --- | --- | --- | --- |
| **Random effects:** | | | | | |
|  | *Groups* | *Name* | *Variance* | *Std.Dev* |  |
|  | Siteyear | (Intercept) | 0.174 | 0.4171 |  |
| **Fixed effects** | | | | | |
| *Name* | | *Estimate* | *Std.Error* | *Z value* | *Pr(>\|z\|)* |
| Intercept | | -3.14 | 0.09 | -34.84 | < 0.001 |
| Standardised Age | | 0.54 | 0.04 | 13.82 | 0.000 |
| Male (vs Female) | | -0.23 | 0.08 | -2.73 | 0.006 |
| Standardised Index of Multiple Deprivation (2004) | | 0.14 | 0.03 | 4.46 | 0.000 |
| Coronary Artery Disease | | 0.25 | 0.08 | 3.36 | 0.001 |
| Heart Failure | | 0.27 | 0.14 | 1.90 | 0.057 |
| COPD | | 0.36 | 0.07 | 4.87 | 0.000 |
| Diabetes | | 0.20 | 0.09 | 2.18 | 0.029 |
| Renal Disease | | 0.44 | 0.13 | 3.52 | 0.000 |
| Cancer | | 0.08 | 0.11 | 0.80 | 0.421 |
| Moderate or Severe Liver Disease | | 3.85 | 0.26 | 14.76 | 0.000 |
| Dyslipidemia | | -0.23 | 0.07 | -3.15 | 0.002 |
| Standardised Annual AAA Volume | | -0.23 | 0.04 | -5.89 | 0.000 |

Adding explanatory variables reduced the estimate of the between year-specific hospital variance (from 0.2988 to 0.174). There is a negative and significant (P-value < 0.001) relationship between volume and in-hospital death for elective Open Repair cases (higher volume associates with lower mortality).

1. CONCLUSIONS

The results from the multi-level modelling approach agree with the results from the single level multivariate modelling approach reported in the main text of the paper. There seems to be negative relationships between volume and in-hospital mortality across all different case-mix groups including emergency EVAR, emergency Open Repair, elective EVAR, and elective Open Repair; however such relationships are only significant for Open Repair (both elective and emergency), they are not significant for EVAR (both elective and emergency).

REFERENCES

Bates, D., et al. (2015). Fitting Linear Mixed-Effects Models Using lme4. *Journal of Statistical Software*.

# **Appendix S3** Intuitive illustration of odds ratio for relationship between volume and in-hospital mortality

The adjusted relationship between volume and in-hospital mortality is reflected by the coefficient of the volume covariate in each model. After case-mix adjustment, a statistically significant volume-in-hospital mortality relationship was seen with emergency OSR (odds ratio, 0.997 ; 95% CI, 0.996 to 0.998; p < 0.01), and elective OSR (odds ratio, 0.996; 95% CI, 0.995 to 0.998; p < 0.01). However, there is no statistically significant relationship even after case-mix adjustment for the emergency EVAR group (odds ratio, 0.999; 95% CI, 0.998 to 1.0004; p = 0.169) and elective EVAR (odds ratio, 0.999; 95% CI, 0.997 to 1.001; p = 0.363). More intuitive illustration of these results (only when statistically significant) are provided below for a patient with the following characteristics: age 74 years old, male, index of multiple deprivation of 20, not ruptured, not weekend admission, admitted in year 2017/18, and no significant comorbidities.

**Table S1** Baseline characteristics between data quintiles

| Clinical Group | Data quintile | % Men | Mean age | mean imd | % ruptured | % weekend admission |
| --- | --- | --- | --- | --- | --- | --- |
|  |  |  |  |  |  |  |
| Emergency EVAR | 1st | 84 | 76.3 | 21.1 | 35 | 16 |
|  | 2nd | 85 | 75.9 | 22.4 | 35 | 17 |
|  | 3rd | 86 | 76.8 | 20.8 | 38 | 15 |
|  | 4th | 82 | 76.6 | 20.6 | 39 | 18 |
|  | 5th | 82 | 75.8 | 19.9 | 44 | 21 |
| Emergency Open Repair | 1st | 82 | 73.4 | 21.3 | 65 | 21 |
|  | 2nd | 82 | 73.9 | 21.6 | 66 | 22 |
|  | 3rd | 82 | 73.7 | 20.3 | 67 | 23 |
|  | 4th | 82 | 73.5 | 21.3 | 66 | 23 |
|  | 5th | 82 | 72.9 | 20.5 | 65 | 22 |
| Elective EVAR | 1st | 89 | 75.4 | 19.5 |  | |
|  | 2nd | 89 | 75.6 | 20.0 |  |  |
|  | 3rd | 89 | 75.6 | 19.8 |  |  |
|  | 4th | 89 | 75.6 | 19.7 |  |  |
|  | 5th | 88 | 75.4 | 19.1 |  |  |
| Elective Open Repair | 1st | 86 | 71.5 | 19.0 |  |  |
|  | 2nd | 85 | 71.4 | 20.1 |  |  |
|  | 3rd | 86 | 71.4 | 19.7 |  |  |
|  | 4th | 86 | 71.2 | 20.3 |  |  |
|  | 5th | 86 | 70.4 | 19.5 |  |  |

| Clinical Group | Data quintile | % year_0607 | % year_0708 | % year_0809 | % year_0910 | % year_1011 | % year_1112 |
| --- | --- | --- | --- | --- | --- | --- | --- |
|  |  |  |  |  |  |  |  |
| Emergency EVAR | 1st | 3% | 4% | 8% | 8% | 9% | 10% |
|  | 2nd | 3% | 7% | 6% | 8% | 12% | 8% |
|  | 3rd | 1% | 1% | 7% | 4% | 11% | 11% |
|  | 4th | 1% | 3% | 2% | 6% | 7% | 8% |
|  | 5th | 3% | 4% | 6% | 9% | 5% | 6% |
| Emergency Open Repair | 1st | 19% | 17% | 14% | 14% | 12% | 10% |
|  | 2nd | 19% | 12% | 12% | 9% | 7% | 9% |
|  | 3rd | 9% | 12% | 11% | 11% | 10% | 9% |
|  | 4th | 8% | 5% | 10% | 5% | 8% | 10% |
|  | 5th | 8% | 10% | 7% | 9% | 6% | 7% |
| Elective EVAR | 1st | 4% | 7% | 9% | 10% | 12% | 12% |
|  | 2nd | 3% | 6% | 9% | 10% | 12% | 10% |
|  | 3rd | 1% | 2% | 5% | 6% | 8% | 10% |
|  | 4th | 2% | 4% | 5% | 5% | 6% | 10% |
|  | 5th | 3% | 4% | 6% | 9% | 6% | 7% |
| Elective Open Repair | 1st | 22% | 17% | 13% | 14% | 11% | 9% |
|  | 2nd | 22% | 16% | 13% | 10% | 7% | 7% |
|  | 3rd | 8% | 13% | 13% | 10% | 10% | 9% |
|  | 4th | 11% | 8% | 9% | 7% | 7% | 6% |
|  | 5th | 10% | 11% | 9% | 7% | 5% | 8% |

| Clinical Group | Data quintile | % year_1213 | % year_1314 | % year_1415 | % year_1516 | % year_1617 | % year_1718 |
| --- | --- | --- | --- | --- | --- | --- | --- |
|  |  |  |  |  |  |  |  |
| Emergency EVAR | 1st | 10% | 11% | 7% | 9% | 11% | 10% |
|  | 2nd | 15% | 6% | 12% | 10% | 3% | 10% |
|  | 3rd | 9% | 10% | 12% | 11% | 17% | 8% |
|  | 4th | 10% | 16% | 11% | 14% | 12% | 9% |
|  | 5th | 8% | 11% | 12% | 9% | 10% | 15% |
| Emergency Open Repair | 1st | 5% | 4% | 3% | 1% | 1% | 1% |
|  | 2nd | 8% | 6% | 5% | 5% | 5% | 3% |
|  | 3rd | 10% | 7% | 6% | 5% | 4% | 5% |
|  | 4th | 11% | 9% | 11% | 8% | 9% | 5% |
|  | 5th | 8% | 10% | 10% | 8% | 8% | 8% |
| Elective EVAR | 1st | 8% | 8% | 8% | 8% | 6% | 7% |
|  | 2nd | 12% | 10% | 6% | 7% | 8% | 7% |
|  | 3rd | 11% | 8% | 14% | 15% | 9% | 9% |
|  | 4th | 11% | 11% | 10% | 11% | 16% | 9% |
|  | 5th | 6% | 15% | 12% | 10% | 10% | 13% |
| Elective Open Repair | 1st | 4% | 3% | 2% | 1% | 1% | 1% |
|  | 2nd | 6% | 5% | 4% | 4% | 4% | 3% |
|  | 3rd | 9% | 6% | 6% | 7% | 4% | 6% |
|  | 4th | 9% | 10% | 10% | 6% | 10% | 6% |
|  | 5th | 6% | 9% | 9% | 7% | 8% | 11% |

| Clinical Group | Data quintile | % Coronary Artery Disease | % Heart Failure | %  COPD | % Diabetes | % Renal Disease | % Cancer | % Moderate or Severe Liver Disease | % Dys-  lipidemia |
| --- | --- | --- | --- | --- | --- | --- | --- | --- | --- |
|  |  |  |  |  |  |  |  |  |  |
| Emergency EVAR | 1st | 26.3 | 7.7 | 28.0 | 14.4 | 8.3 | 9.1 | 0.3 | 29.2 |
|  | 2nd | 26.6 | 8.9 | 27.5 | 14.1 | 7.9 | 8.2 | 0.8 | 24.9 |
|  | 3rd | 25.5 | 7.8 | 27.9 | 15.0 | 9.3 | 9.8 | 0.6 | 25.2 |
|  | 4th | 26.0 | 8.6 | 29.4 | 15.7 | 8.3 | 9.8 | 0.3 | 30.1 |
|  | 5th | 25.0 | 8.0 | 33.2 | 15.0 | 8.7 | 8.4 | 0.3 | 30.7 |
| Emergency Open Repair | 1st | 15.5 | 3.7 | 21.3 | 12.1 | 3.5 | 5.2 | 1.8 | 21.0 |
|  | 2nd | 14.7 | 3.2 | 20.6 | 10.7 | 3.3 | 5.6 | 1.0 | 20.4 |
|  | 3rd | 16.1 | 3.9 | 21.0 | 11.6 | 4.0 | 5.2 | 1.7 | 21.0 |
|  | 4th | 15.8 | 4.0 | 22.6 | 11.4 | 4.3 | 4.9 | 1.9 | 20.3 |
|  | 5th | 15.1 | 3.3 | 22.6 | 12.6 | 4.3 | 5.8 | 1.0 | 24.4 |
| Elective EVAR | 1st | 28.9 | 5.6 | 26.2 | 16.5 | 7.1 | 12.8 | 0.3 | 30.0 |
|  | 2nd | 28.4 | 7.0 | 26.2 | 16.9 | 8.4 | 13.4 | 0.4 | 31.8 |
|  | 3rd | 28.6 | 6.4 | 26.3 | 17.4 | 9.1 | 13.1 | 0.4 | 32.4 |
|  | 4th | 30.2 | 7.4 | 27.6 | 17.3 | 7.9 | 13.4 | 0.3 | 36.2 |
|  | 5th | 32.2 | 7.7 | 27.6 | 18.4 | 9.8 | 14.4 | 0.4 | 39.6 |
| Elective Open Repair | 1st | 23.3 | 3.9 | 18.5 | 13.0 | 2.9 | 9.3 | 0.3 | 28.1 |
|  | 2nd | 24.7 | 4.0 | 19.5 | 12.4 | 4.6 | 8.7 | 0.5 | 28.1 |
|  | 3rd | 21.9 | 4.2 | 19.9 | 12.4 | 4.2 | 8.1 | 0.5 | 30.3 |
|  | 4th | 22.3 | 3.6 | 19.2 | 12.9 | 3.9 | 9.0 | 0.3 | 34.1 |
|  | 5th | 22.5 | 4.7 | 19.2 | 12.0 | 6.3 | 7.7 | 0.2 | 36.6 |

**Table S2** Fixed-effect logistic regression model of in-hospital mortality. Emergency EVAR

| **Covariate** | **Coefficient** | | **OR  (exp coef)** | **95% CI OR** | | **P-value** |
| --- | --- | --- | --- | --- | --- | --- |
|  |  |  |  | **LCI** | **UCI** |  |
| **Intercept** | | -5.57913 | 0.00378 | 0.00124 | 0.01108 | 0.00000 |
| **Age in years** | | 0.04018 | 1.04100 | 1.02948 | 1.05288 | 0.00000 |
| **Male (vs Female)** | | -0.28829 | 0.74955 | 0.60573 | 0.93158 | 0.00862 |
| **Index of Multiple Deprivation (2004)** | | 0.00247 | 1.00247 | 0.99676 | 1.00812 | 0.39247 |
| **Ruptured AAA** | | 1.75320 | 5.77303 | 4.82047 | 6.94234 | 0.00000 |
| **Admitted in weekend (Saturday or Sunday)** | | 0.18844 | 1.20737 | 0.98116 | 1.47924 | 0.07182 |
| **HESYEAR in the data (200607 as reference)** | |  |  |  |  |  |
| hesyear200708 | | 0.07817 | 1.08130 | 0.53855 | 2.24189 | 0.82897 |
| hesyear200809 | | -0.16676 | 0.84641 | 0.44015 | 1.69735 | 0.62628 |
| hesyear200910 | | 0.01443 | 1.01453 | 0.54301 | 1.99048 | 0.96507 |
| hesyear201011 | | 0.01875 | 1.01893 | 0.55184 | 1.98110 | 0.95389 |
| hesyear201112 | | -0.06495 | 0.93712 | 0.50807 | 1.82020 | 0.84099 |
| hesyear201213 | | -0.41143 | 0.66270 | 0.35766 | 1.29118 | 0.20643 |
| hesyear201314 | | -0.45597 | 0.63383 | 0.34269 | 1.23323 | 0.16041 |
| hesyear201415 | | -0.12287 | 0.88438 | 0.48556 | 1.70083 | 0.69918 |
| hesyear201516 | | -0.32178 | 0.72486 | 0.39348 | 1.40622 | 0.31926 |
| hesyear201617 | | -0.14136 | 0.86818 | 0.47323 | 1.67917 | 0.65993 |
| hesyear201718 | | -0.28058 | 0.75535 | 0.40996 | 1.46542 | 0.38522 |
| **Presence of cormobidities** | |  |  |  |  |  |
| Coronary Artery Disease | | -0.03599 | 0.96465 | 0.77646 | 1.19353 | 0.74270 |
| Heart Failure | | 0.04322 | 1.04417 | 0.75435 | 1.42968 | 0.79075 |
| COPD | | 0.29040 | 1.33696 | 1.11386 | 1.60202 | 0.00173 |
| Diabetes | | -0.11532 | 0.89108 | 0.68920 | 1.14118 | 0.36952 |
| Renal Disease | | 0.34207 | 1.40786 | 1.05297 | 1.86594 | 0.01898 |
| Cancer | | -0.19005 | 0.82692 | 0.59868 | 1.12241 | 0.23509 |
| Moderate or Severe Liver Disease | | 1.78852 | 5.98057 | 2.36525 | 14.37599 | 0.00009 |
| Dyslipidemia | | -0.23011 | 0.79445 | 0.64610 | 0.97247 | 0.02728 |
| **Annual AAA Volume** | | -0.00095 | 0.99905 | 0.99770 | 1.00039 | 0.16899 |

**Table S3** Fixed-effect logistic regression model of in-hospital mortality. Emergency OSR

| **Covariate** | **Coefficient** | **OR  (exp coef)** | **95% CI OR** | | | **P-value** | |  |
| --- | --- | --- | --- | --- | --- | --- | --- | --- |
|  |  |  | **LCI** | | **UCI** |  |  |  |
| **Intercept** | -6.16187 | 0.00211 | 0.00138 | 0.00322 | | | 0.00000 | |
| **Age in years** | 0.06170 | 1.06364 | 1.05833 | 1.06903 | | | 0.00000 | |
| **Male (vs Female)** | -0.16735 | 0.84590 | 0.76952 | 0.93020 | | | 0.00054 | |
| **Index of Multiple Deprivation (2004)** | 0.00508 | 1.00509 | 1.00261 | 1.00758 | | | 0.00006 | |
| **Ruptured AAA** | 1.34799 | 3.84969 | 3.51794 | 4.21733 | | | 0.00000 | |
| **Admitted in weekend (Saturday or Sunday)** | 0.14962 | 1.16139 | 1.06558 | 1.26547 | | | 0.00065 | |
| **HESYEAR in the data (200607 as reference)** |  |  |  |  | | |  | |
| hesyear200708 | -0.07429 | 0.92840 | 0.80005 | 1.07716 | | | 0.32739 | |
| hesyear200809 | -0.04679 | 0.95429 | 0.82112 | 1.10888 | | | 0.54148 | |
| hesyear200910 | -0.14858 | 0.86193 | 0.73670 | 1.00800 | | | 0.06319 | |
| hesyear201011 | -0.24822 | 0.78019 | 0.66331 | 0.91706 | | | 0.00266 | |
| hesyear201112 | -0.25907 | 0.77177 | 0.65640 | 0.90684 | | | 0.00167 | |
| hesyear201213 | -0.17942 | 0.83575 | 0.70861 | 0.98507 | | | 0.03272 | |
| hesyear201314 | 0.01936 | 1.01954 | 0.85884 | 1.20958 | | | 0.82462 | |
| hesyear201415 | 0.04218 | 1.04309 | 0.87440 | 1.24351 | | | 0.63860 | |
| hesyear201516 | 0.02127 | 1.02150 | 0.84503 | 1.23344 | | | 0.82543 | |
| hesyear201617 | 0.03863 | 1.03938 | 0.85884 | 1.25646 | | | 0.69056 | |
| hesyear201718 | -0.04726 | 0.95383 | 0.77345 | 1.17394 | | | 0.65691 | |
| **Presence of cormobidities** |  |  |  |  | | |  | |
| Coronary Artery Disease | 0.19081 | 1.21023 | 1.08630 | 1.34765 | | | 0.00052 | |
| Heart Failure | 0.20472 | 1.22718 | 1.00546 | 1.49581 | | | 0.04327 | |
| COPD | 0.24779 | 1.28118 | 1.17333 | 1.39860 | | | 0.00000 | |
| Diabetes | 0.14188 | 1.15244 | 1.02864 | 1.29020 | | | 0.01408 | |
| Renal Disease | 0.55511 | 1.74213 | 1.44578 | 2.09867 | | | 0.00000 | |
| Cancer | 0.07887 | 1.08207 | 0.92126 | 1.26879 | | | 0.33391 | |
| Moderate or Severe Liver Disease | 3.05848 | 21.29515 | 13.78128 | 34.52676 | | | 0.00000 | |
| Dyslipidemia | -0.13352 | 0.87501 | 0.79579 | 0.96159 | | | 0.00568 | |
| **Annual AAA Volume** | -0.00313 | 0.99688 | 0.99617 | 0.99758 | | | 0.00000 | |

**Table S4** Fixed-effect logistic regression model of of in-hospital mortality. Elective EVAR

| **Covariate** | **Coefficient** | | **OR  (exp coef)** | **95% CI OR** | | | **P-value** | |  |
| --- | --- | --- | --- | --- | --- | --- | --- | --- | --- |
|  |  |  |  | **LCI** | | **UCI** |  |  |  |
| **Intercept** | | -6.50069 | 0.00150 | 0.00036 | 0.00606 | | | 0.00000 | |
| **Age in years** | | 0.03662 | 1.03730 | 1.02104 | 1.05412 | | | 0.00001 | |
| **Male (vs Female)** | | -0.67383 | 0.50975 | 0.39086 | 0.67335 | | | 0.00000 | |
| **Index of Multiple Deprivation (2004)** | | 0.00528 | 1.00530 | 0.99805 | 1.01233 | | | 0.14435 | |
| **HESYEAR in the data (200607 as reference)** | |  |  |  |  | | |  | |
| hesyear200708 | | 0.26401 | 1.30214 | 0.69488 | 2.58719 | | | 0.42711 | |
| hesyear200809 | | -0.19368 | 0.82392 | 0.43815 | 1.64067 | | | 0.56184 | |
| hesyear200910 | | -0.22008 | 0.80246 | 0.43248 | 1.58379 | | | 0.50262 | |
| hesyear201011 | | -0.48603 | 0.61506 | 0.32700 | 1.22497 | | | 0.14559 | |
| hesyear201112 | | -0.48022 | 0.61865 | 0.33323 | 1.22153 | | | 0.14383 | |
| hesyear201213 | | -0.62106 | 0.53737 | 0.28268 | 1.07768 | | | 0.06643 | |
| hesyear201314 | | -0.34062 | 0.71133 | 0.38743 | 1.39460 | | | 0.29312 | |
| hesyear201415 | | -0.60434 | 0.54643 | 0.28966 | 1.09097 | | | 0.07155 | |
| hesyear201516 | | -1.08182 | 0.33898 | 0.16863 | 0.70496 | | | 0.00275 | |
| hesyear201617 | | -0.94829 | 0.38740 | 0.19550 | 0.79856 | | | 0.00766 | |
| hesyear201718 | | -0.77138 | 0.46237 | 0.23720 | 0.94345 | | | 0.02710 | |
| **Presence of cormobidities** | |  |  |  |  | | |  | |
| Coronary Artery Disease | | 0.47443 | 1.60710 | 1.27538 | 2.01956 | | | 0.00005 | |
| Heart Failure | | 0.15969 | 1.17314 | 0.80453 | 1.66474 | | | 0.38806 | |
| COPD | | 0.41119 | 1.50862 | 1.20171 | 1.88580 | | | 0.00034 | |
| Diabetes | | 0.05465 | 1.05617 | 0.79309 | 1.38609 | | | 0.70076 | |
| Renal Disease | | 0.46682 | 1.59491 | 1.15844 | 2.15577 | | | 0.00316 | |
| Cancer | | 0.05679 | 1.05843 | 0.77196 | 1.42152 | | | 0.71482 | |
| Moderate or Severe Liver Disease | | 2.70979 | 15.02616 | 8.09357 | 26.18656 | | | 0.00000 | |
| Dyslipidemia | | -0.15334 | 0.85784 | 0.67707 | 1.08099 | | | 0.19844 | |
| **Annual AAA Volume** | | -0.00085 | 0.99915 | 0.99729 | 1.00096 | | | 0.36319 | |

**Table S5** Fixed-effect logistic regression model of of in-hospital mortality. Elective OSR

| **Covariate** | **Coefficient** | | **OR  (exp coef)** | **95% CI OR** | | | **P-value** | |  |
| --- | --- | --- | --- | --- | --- | --- | --- | --- | --- |
|  |  |  |  | **LCI** | | **UCI** |  |  |  |
| **Intercept** | | -7.32986 | 0.00066 | 0.00031 | 0.00137 | | | 0.00000 | |
| **Age in years** | | 0.06246 | 1.06446 | 1.05483 | 1.07430 | | | 0.00000 | |
| **Male (vs Female)** | | -0.22809 | 0.79605 | 0.67900 | 0.93717 | | | 0.00551 | |
| **Index of Multiple Deprivation (2004)** | | 0.00979 | 1.00984 | 1.00571 | 1.01392 | | | 0.00000 | |
| **HESYEAR in the data (200607 as reference)** | |  |  |  |  | | |  | |
| hesyear200708 | | 0.02945 | 1.02988 | 0.82474 | 1.28493 | | | 0.79445 | |
| hesyear200809 | | 0.13634 | 1.14608 | 0.91594 | 1.43260 | | | 0.23178 | |
| hesyear200910 | | -0.00049 | 0.99951 | 0.78102 | 1.27425 | | | 0.99688 | |
| hesyear201011 | | -0.08796 | 0.91580 | 0.69843 | 1.19220 | | | 0.51851 | |
| hesyear201112 | | 0.00084 | 1.00084 | 0.76452 | 1.30154 | | | 0.99506 | |
| hesyear201213 | | -0.08828 | 0.91550 | 0.68094 | 1.21776 | | | 0.55102 | |
| hesyear201314 | | -0.17379 | 0.84047 | 0.61297 | 1.13681 | | | 0.26922 | |
| hesyear201415 | | -0.28078 | 0.75520 | 0.53527 | 1.04634 | | | 0.09989 | |
| hesyear201516 | | -0.04587 | 0.95516 | 0.67961 | 1.32003 | | | 0.78611 | |
| hesyear201617 | | -0.46985 | 0.62510 | 0.41735 | 0.90875 | | | 0.01759 | |
| hesyear201718 | | -0.16787 | 0.84547 | 0.58636 | 1.19346 | | | 0.35340 | |
| **Presence of cormobidities** | |  |  |  |  | | |  | |
| Coronary Artery Disease | | 0.25060 | 1.28479 | 1.10842 | 1.48587 | | | 0.00080 | |
| Heart Failure | | 0.26933 | 1.30908 | 0.99282 | 1.70252 | | | 0.04998 | |
| COPD | | 0.36928 | 1.44669 | 1.25175 | 1.66772 | | | 0.00000 | |
| Diabetes | | 0.20596 | 1.22871 | 1.02921 | 1.45864 | | | 0.02053 | |
| Renal Disease | | 0.48006 | 1.61618 | 1.26262 | 2.04548 | | | 0.00009 | |
| Cancer | | 0.07542 | 1.07834 | 0.87377 | 1.31875 | | | 0.47222 | |
| Moderate or Severe Liver Disease | | 3.77554 | 43.62115 | 26.83590 | 72.48002 | | | 0.00000 | |
| Dyslipidemia | | -0.23174 | 0.79315 | 0.68580 | 0.91509 | | | 0.00163 | |
| **Annual AAA Volume** | | -0.00362 | 0.99639 | 0.99514 | 0.99761 | | | 0.00000 | |
